# Editorial: Crosstalk of Mitochondria With Brain Insulin and Leptin Signaling

**DOI:** 10.3389/fendo.2018.00761

**Published:** 2018-12-14

**Authors:** André Kleinridders, Heather A. Ferris, Sulay Tovar

**Affiliations:** ^1^Central Regulation of Metabolism, German Institute of Human Nutrition Potsdam-Rehbruecke, Nuthetal, Germany; ^2^German Center for Diabetes Research (DZD), Neuherberg, Germany; ^3^Division of Endocrinology and Metabolism, University of Virginia, Charlottesville, VA, United States; ^4^Departamento de Fisioloxía, Centro de Investigación en Medicina Molecular (CIMUS), Instituto de Investigaciones Sanitarias de Santiago de Compostela (IDIS), Universidade de Santiago de Compostela, Santiago de Compostela, Spain; ^5^CIBER Fisiopatología de la Obesidad y Nutrición (CIBERobn), Madrid, Spain

**Keywords:** insulin, leptin, brain, mitochondria, chaperones

The world is facing an obesity epidemic and, with it, an increase of co-morbidities such as type 2 diabetes (T2D). Obesity can be considered in part as a brain disease, characterized by an imbalance of energy intake and energy expenditure. This interplay is mainly controlled by leptin and insulin action in the hypothalamus, ensuring proper weight regulation. Although dietary interventions and guidelines have been in place for decades, long-term weight reduction is difficult to maintain because of compensatory hormonal mechanisms favoring weight regain (1, 2). Obese and T2D patients are characterized by insulin and leptin resistance in the brain, a state where the organism exhibits an inadequate response to these hormones resulting in hyperglycemia, hyperphagia, decreased energy expenditure and obesity. Moreover, the brain of T2D patients exhibits oxidative stress and mitochondrial dysfunction which is linked to structural abnormalities and altered connectivity (3).

The brain coordinates overall body function, ranging from modulating adaptive responses to different metabolic stimuli to implementing proper behavioral actions. To do so, the brain consumes enormous amounts of energy, mainly in the form of glucose, and converts it into energy in the form of ATP via oxidative phosphorylation in mitochondria.

Therefore, deterioration of mitochondrial function impacts behavior and contributes to obesity. Mitochondrial dysfunction can cause insulin resistance in the hypothalamus (4). Reciprocally, leptin resistance causes brain mitochondrial dysfunction and concomitantly induces insulin resistance, further exemplifying the close interaction of mitochondria, leptin and insulin signaling in the brain (4). Mice which lack leptin receptors in the brain are insulin resistant, whereas mice lacking insulin receptor in the brain are obese and hyperleptinemic, signs of leptin resistance (5, 6). Thus, understanding how the underlying mechanisms of insulin and leptin resistance modify mitochondrial function are crucial to identify novel targeting strategies to combat obesity and its co-morbidities. To this end, concepts of leptin resistance are reviewed in this Research Topic, shedding light onto possible explanations for the occurrence of leptin resistance. The novel concept of redox-based leptin signaling, the NSAPP pathway, is here of special interest, as it provides an explanation why mild oxidizing agents are important for proper leptin and insulin signaling in the hypothalamus, a phenomenon which was first observed almost 30 years ago (7). This pathway consists of an oxide transport chain mediated by NOX3 and SOD3 that generates a transient H_2_O_2_ burst which suppresses leptin-inhibiting phosphatases (Fruhwürth et al.). This pathway is crucial for leptin and insulin action in the brain, and represents a novel target to combat the metabolic consequences of insulin resistance, such as obesity and hyperglycemia.

Hyperglycemia is a feature of insulin resistance and T2D. As glucose uptake in the brain is mainly insulin-independent, brain mitochondria have to adapt quickly to different nutrient supplies. This is facilitated by changing mitochondrial dynamics, increasing their number or improving their function via post-translational protein modifications, adaptations which deteriorate in metabolic disorders. Furthermore, mitochondrial dynamics are important for glucose and leptin-sensing POMC neurons, underlining again the crosstalk of mitochondria and leptin signaling as well as their consequences on insulin action (8).

Thus, Cunarro and colleagues review this crosstalk and propose hypothalamic mitochondrial function as a target to attenuate hyperphagia, thereby reversing obesity and diabetes (Cunarro et al.). Another hypothesis has been postulated in this Research Topic, which states that mitochondrial chaperones can improve mitochondrial function and thereby brain function. This is of special interest, as impaired expression of many mitochondrial chaperones is linked to neurodegeneration (Castro et al.) and mitochondrial dysfunction is linked to insulin and leptin resistance. Understanding the regulation of these chaperones might pave the way for novel therapeutic approaches for brain diseases.

Besides the well-known effects of insulin and leptin on metabolism, both hormones are involved in cognition and behavior. T2D patients are at increased risk of developing neurodegenerative diseases, suggesting a link of insulin and leptin resistance with cognitive impairment and neurodegeneration. While the links between hormone resistance and mitochondrial dysfunction are robust, the mechanisms linking these to cognitive function remain controversial. Therapeutic interventions aimed at reducing insulin resistance in humans have had so far limited success in modifying the progression of cognitive decline. Insulin sensitizers can attenuate cognitive decline in obese, insulin-resistant animals, and use of insulin-sensitizers or intranasal insulin treatment have been shown to improve cognition in small sub-cohorts of Alzheimer's (AD) patients. Yet, not all AD patients benefit from these interventions (9, 10). Interestingly, in these unresponsive patients, mitochondrial damage may be more important for their cognitive decline, suggesting that mitochondrial dysfunction cannot easily be compensated for by administration of insulin or insulin sensitizers (11). More research is needed to better understand the disease progression and identify patient groups who will optimally respond to treatment regimens (12). To this end, one review article summarizes the link of insulin resistance, mitochondrial dysfunction and cognitive decline, discussing controversial observations and reporting on current and novel strategies to combat these diseases (Sripetchwandee et al.).

Overall, the review articles in this Research Topic highlight the crosstalk of key metabolic hormones with mitochondria and their impact on metabolism and behavior. The articles not only focus on mechanistic insights and metabolic outcomes, but also present hypotheses about the development of hormone resistance in the brain and novel concepts to improve brain function and health.

## Author Contributions

AK conceptualized and wrote the manuscript. HF wrote and edited the manuscript. ST edited the manuscript. All authors edited and approved the final version of this manuscript.

### Conflict of Interest Statement

The authors declare that the research was conducted in the absence of any commercial or financial relationships that could be construed as a potential conflict of interest.
